# Melatonin increases overall survival of prostate cancer patients with poor prognosis after combined hormone radiation treatment

**DOI:** 10.18632/oncotarget.27757

**Published:** 2020-10-13

**Authors:** Gennady M. Zharinov, Oleg A. Bogomolov, Irina V. Chepurnaya, Natalia Yu. Neklasova, Vladimir N. Anisimov

**Affiliations:** ^1^A.M. Granov Russian Research Center for Radiology and Surgical Technologies of the Ministry of Health of the Russian Federation, Pesochny, St. Petersburg 197758, Russia; ^2^N.N. Petrov National Medical Research Center of Oncology, Pesochny, St. Petersburg 197758, Russia

**Keywords:** prostate cancer, overall survival, poor prognosis, melatonin

## Abstract

Background: The antitumor and immunomodulating activities of melatonin are widely known. These activities are based upon the multifactorial mechanism of action on various links of carcinogenesis. In the present paper, the long-term results of the clinical use of melatonin in the combined treatment of patients with prostate cancer of various risk groups were evaluated.

Materials and Methods: A retrospective study included 955 patients of various stages of prostate cancer (PCa) who received combined hormone radiation treatment from 2000 to 2019. Comprehensive statistical methods were used to analyze the overall survival rate of PCa patients treated with melatonin in various prognosis groups.

Results: The overall survival rate of PCa patients with favorable and intermediate prognoses treated or not treated with melatonin was not statistically significantly different. In the poor prognosis group, the median overall survival in patients taking the drug was 153.5 months versus 64.0 months in patients not using it (*p* < 0.0001). The 5-year overall survival rates in the research and control groups were 66.8 ± 1.9 and 53.7 ± 2.6 (*p* < 0.0001) respectively. In a multivariate analysis, melatonin administration proved to be an independent prognostic factor and reduced the risk of death of PCa patients by more than twice (*p* < 0.0001).

Conclusions: The multicomponent antitumor effect of melatonin is fully realized and clearly demonstrated in treatment of PCa patients with poor prognosis with a set of unfavorable factors of the tumor progression.

## INTRODUCTION

Melatonin is the main hormone of the pineal gland. It is a regulator of the circadian rhythm of all living organisms and has antioxidant, immunomodulating, and antitumor activity [1, 2]. The antitumor effect of melatonin has been shown both *in vitro* and *in vivo* in a number of experimental models in rodents. Localizations include the mammary gland, colon, uterine, cervix, lung, etc [3–5]. Melatonin is most widely used in clinical practice to treat hormone-dependent tumors, and primarily in the combined treatment of breast cancer [6]. According to available data, the use of melatonin led to an increase in the number of objective responses and/or overall survival of patients, and was accompanied by a decrease in the frequency of side effects of drug antitumor and radiation therapy [1, 6, 7].

Some possible points of inhibition of tumor growth by melatonin included activation of T helper type 1; increased production of several cytokines (IL-2, IFN-γ, IL-6); inhibition of angiogenesis; reduced expression of the VEGF receptors; activation of apoptosis in tumor cells; and a decrease in telomerase activity [1, 6, 8–10].

Prostate cancer (PCa) is the second most frequently occurring neoplasia and the fifth leading cause of cancer mortality in men [11]. Night shift work and light at night induced circadian disruption, followed by a decline in the circulating melatonin level, which is one of possible cause of high risk of PCa [12, 13]. Nevertheless, the data on the role of melatonin in prevention and treatment of PCa is rather scarce.

A number of epidemiological studies have shown the clinical effectiveness of melatonin in reducing both the risk of developing PCa and its aggressiveness [1, 14]. *In vitro/in vivo* studies demonstrate various mechanisms of inhibition of tumor proliferation of prostate adenocarcinoma cells both by enhancing apoptosis and cytoreduction, and by reducing tumor potential and angiogenesis [6, 7, 15, 16]. Of particular interest are the works demonstrating positive results of melatonin use in treatment of castrate-resistant metastatic PCa, when the possibilities of most available drugs have already been exhausted. Thus, in the study of P. Lissoni et al. [17, 18] the results of the use of melatonin drugs in palliative treatment of patients with end-stage prostate cancer are shown. Of the four patients, three men were observed to have stabilized the disease, which allowed them to overcome the two-year threshold of overall survival.

Thus, the assessment of long-term results of wide clinical use of melatonin in the combined treatment of patients with prostate cancer of various risk groups is an urgent and relevant task.

The aim of the study was to improve the effectiveness of treatment of patients with prostate cancer by long-term use of melatonin drugs after a course of combined hormone-radiation treatment.

## RESULTS

**Figure 1:**
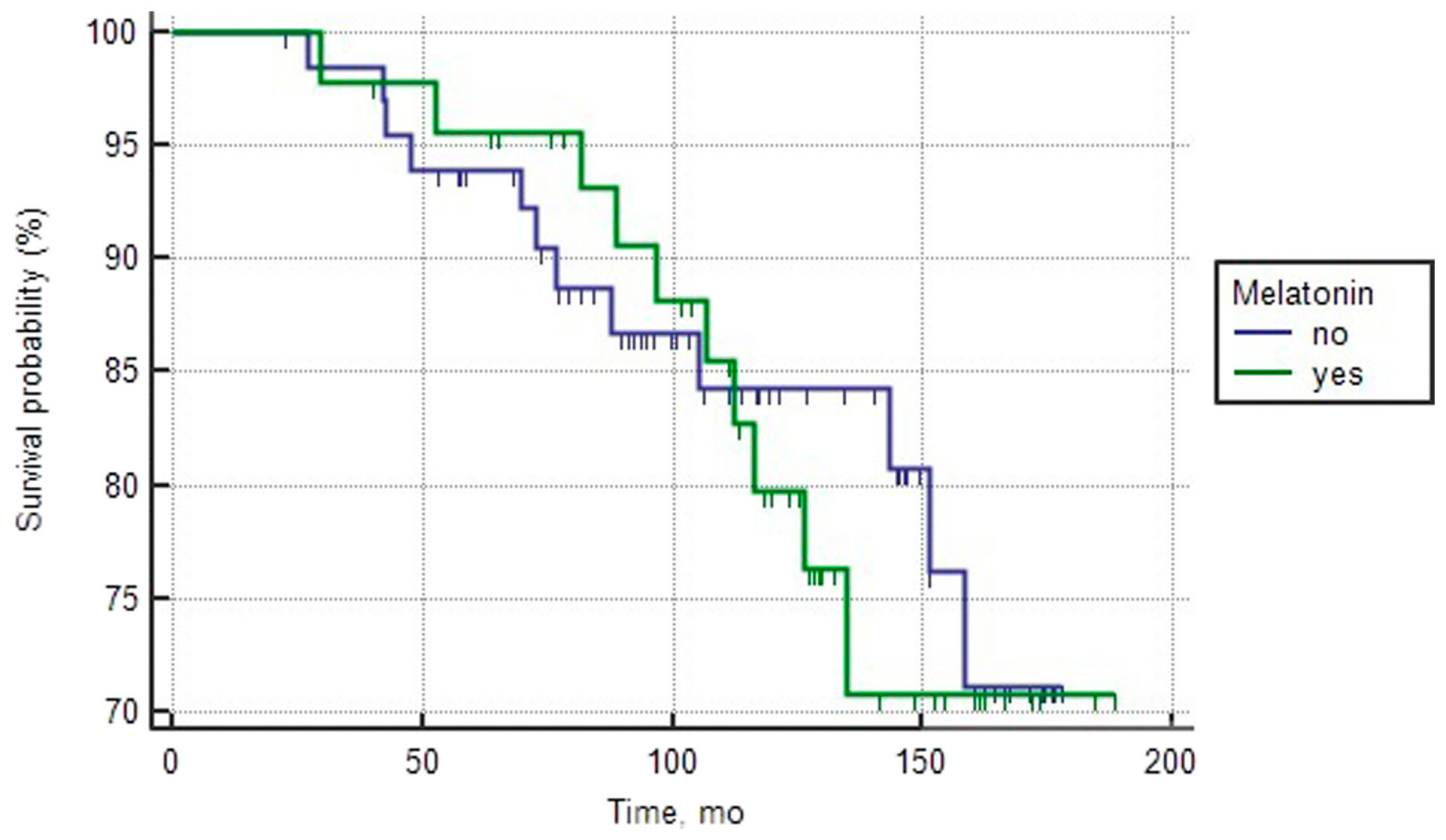
Overall survival curves of patients with good prognosis PCa depending on the intake of melatonin (log rank test > 0.05).

**Figure 2:**
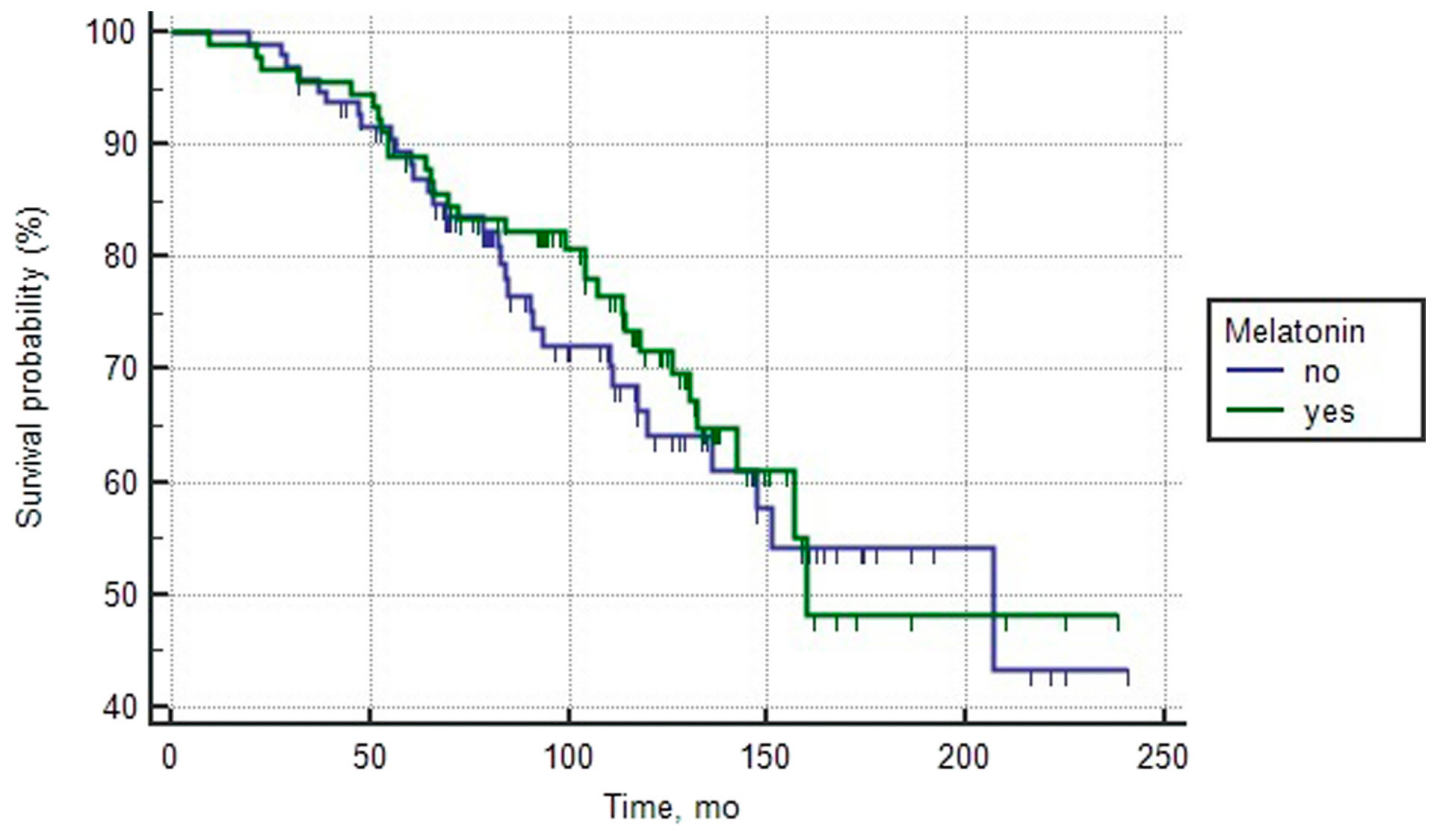
Overall survival curves of patients with intermediate prognosis PCa depending on the intake of melatonin (log rank test > 0.05).

The analysis of both groups of patients with favorable prognosis of PCa as well as both groups with intermediate prognosis, which were treated or not treated with melatonin, revealed both similar basic clinical and morphological characteristics, as well as the median overall survival (Tables 1 and 2). Single-factor analysis and log-rank test did not show a statistically significant effect of melatonin intake on survival rates (Figures 1 and 2).

**Table 1:** Clinical characteristics of favorable prognosis prostate cancer patients (*n* = 113)

Parameters	Patients treated with Melatonin	Patients treated without Melatonin	*p*
Number	46	67	
Age at diagnosis, years, Me (IQR)	66.1 (59.3–70.5)	63.1 (59.5–70.7)	> 0.05
PSA at diagnosis, ng/ml, Me (IQR)	6.9 (5.6–8.7)	6.7 (4.5–8.6)	> 0.05
EBRT dose, Gy, Me (IQR)	68.0 (66.0–70.0)	68.5 (66.0–70.5)	> 0.05
Time of follow up, mo, Me (IQR)	125.9 (101.6–148.5)	106.0 (76.6–151.2)	> 0.05
Overall survival, Mean ± SE	162.9 ± 7.1	157.2 ± 5.5	> 0.05
10-Y Overall survival, %	79.7	84.2	> 0.05

**Table 2:** Clinical characteristics of intermediate prognosis prostate cancer patients (*n* = 187)

Parameters	Patients treated with Melatonin	Patients treated without Melatonin	*p*
Number	91	96	
Age at diagnosis, years, Me (IQR)	67.0 (61.3–79.6)	64.6 (60.7–67.9)	> 0.05
PSA at diagnosis, ng/ml, Me (IQR)	16.0 (11.9–18.9)	15.5 (12.5–18.6)	> 0.05
EBRT dose, Gy, Me (IQR)	68.5 (66.5–70.5)	68.5 (66.0–71.0)	> 0.05
Time of follow up, mo, Me (IQR)	110.0 (92.4–136.7)	93.5 (68.6–128.6)	> 0.05
Overall survival, Mean ± SE	170.0.9 ± 7.2	167.9 ± 10.1	> 0.05
10-Y Overall survival, %	68.3	64.1	> 0.05

**Figure 3:**
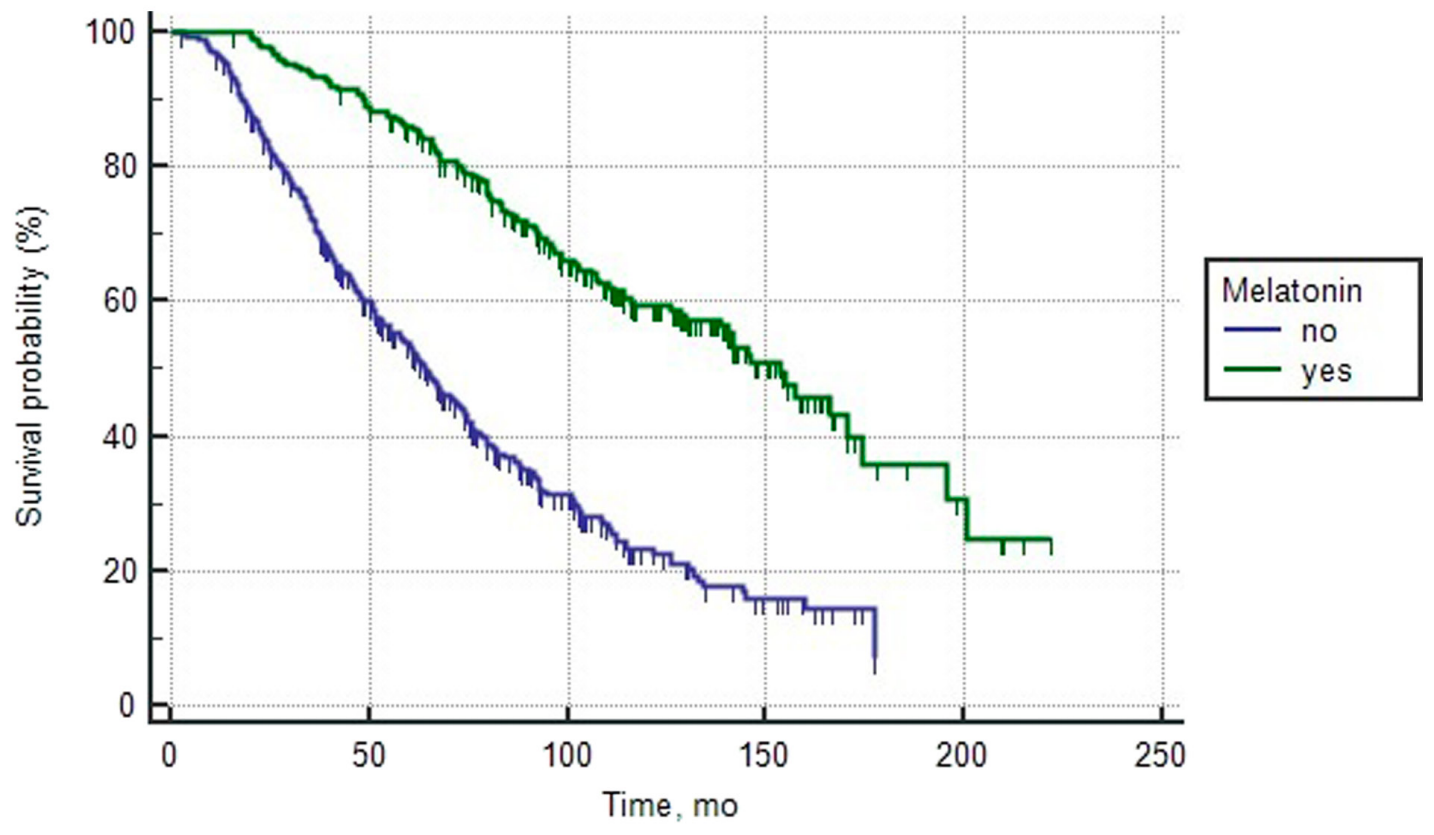
Overall survival curves of patients with poor prognosis PCa depending on the intake of melatonin (log rank test < 0.0001).

The general clinical and morphological characteristics of patients with poor prognosis of prostate cancer are presented in Table 3. Single-factor analysis showed that the initial PSA level, the sum of Gleason scores, melatonin intake, the presence of clinically positive regional lymph nodes, the pretreatment PSA doubling time (PSADT), and the number of metastatic foci localities had a significant impact on overall survival. In order to decimate their independent predictive significance, a multi-factor analysis of proportional Cox risks was performed, and the method of forced entry was used. The multivariate model included the following quantitative (pretreatment PSA and PSADT) and categorical independent factors: clinically positive pelvic lymph nodes (N0/N1); Gleason score lowGS (6–7)/medGS (8)/ highGS (9–10); melatonin intake (yes/no), and the number of metastatic foci localities (1site/2sites/3sites). The results of the multi-factor analysis are presented in Table 4. Inclusion of the morphological factors in a multivariate model revealed that independent predictors of risk of death was Gleason sum by biopsy 8 and 9–10, the presence of distant metastases, and lack of intake of Melatonin. The most powerful influence on the decrease of survival rates was naturally exerted by the following worse factors: the presence of three localities of metastatic foci (Ma+b+c), as well as the sum of Gleason scores of 9–10 (Exp (b) = 5.2 and 3.0, respectively). The risk of death in patients with PCa was more than double in the presence of two metastases and the absence of Melatonin intake (Exp (b) = 2.5 and 2.2, respectively). Figure 3 shows the overall survival curves of the control and research groups of patients with poor prognosis of PCa, formed depending on the intake of Melatonin. The median overall survival in patients treated with the drug was 153.5 months compared to 64.0 months in the patients who were not treated with the drugs. The 5-year overall survival rates in the melatonin-treated and control groups were 66.8 ± 1.9 and 53.7 ± 2.6, respectively.

**Table 3:** Clinical and morphological characteristics of poor prognosis prostate cancer patients (*n* = 655)

Parameters	Patients treated with Melatonin	Patients treated without Melatonin	*p*
Number	259	396	
Age at diagnosis, years, Me (IQR)	65.0 (58.8–71.7)	64.5 (59.6–69.4)	> 0.05
PSA at diagnosis, ng/ml, Me (IQR)	33.5 (19.0–53.0)	43.9 (29.9–85.6)	< 0.01
Pretreatment PSADT, mo, Me (IQR)	21.7 (8.2–48.8)	16.0 (4.6–38.7)	< 0.05
Biopsy Gleason score, *n* (%)			
6–7	126 (48.6)	180 (45.5)	> 0.05
8	112 (43.2)	153 (38.6)	> 0.05
9–10	21 (8.1)	63 (15.9)	< 0.01
Clinical positive lymphnodes, *n* (%)	69 (26.6)	141 (35.6)	< 0.05
Number of metastatic sites, *n* (%)			
1 (M1a or 1b or 1c)	110 (42.5)	196 (49.5)	> 0.05
2 (M1a+b or 1a+c or 1b+c)	9 (3.5)	24 (6.1)	< 0.01
3 (M1a+b+c)	6 (2.3)	12 (3.0)	> 0.05
5-Y Overall survival, %	66.8 ± 1.9	53.7 ± 2.6	< 0.0001

**Table 4:** Results of multivariate analysis

Covariate	b	SE	Wald	*p*	Exp (b)	95% CI of Exp (b)
medGS (8)	0.3897	0.1469	7.0348	0.0080	1.4766	1.1071 to 1.9694
highGS (9–10)	1.1064	0.1998	30.6510	< 0.0001	3.0234	2.0436 to 4.4731
1site mts	0.4583	0.1474	9.6705	0.0019	1.5814	1.1847 to 2.1111
2 sites mts	0.9099	0.4022	5.1176	0.0237	2.4841	1.1293 to 5.4644
3 sites mts	1.6538	0.4451	13.8055	0.0002	5.2266	2.1845 to 12.5052
Melatonin – no	0.7932	0.1449	29.9746	< 0.0001	2.2105	1.6640 to 2.9364

## DISCUSSION

The analysis of the overall survival of patients with PCa with both favorable and intermediate prognosis did not reveal any significant differences depending on the long-term use of melatonin drugs. These categories of patients after radical treatment had high rates of cancer effectiveness and were characterized by long-term overall survival (median overall survival was not achieved in any of the groups with an average follow-up period of more than 10 years). The fact that the modern methods of radiation and hormone therapy in patients with local and highly-differentiated PCa is very effective, completely suppresses the tumor process, providing excellent clinical results, and may explain the lack of significant influence of melatonin on the life expectancy of these patients. When a complete local response is achieved against the background of the treatment, there are physically no conditions and application points for the implementation of the antitumor effect of melatonin.

In the group of patients with poor prognosis, we see the opposite picture. Multi-sided statistical analysis demonstrated a clear positive effect of melatonin administration on long-term survival rates. At a 5-year median follow-up, patients who received melatonin had an overall survival rate that, on average, was 13 months longer as compared to the control group. This conclusion was proven by a multi-factor analysis, where treatment with melatonin served as an independent predictive factor and reduced the risk of death in patients with PCa by more than 2 times.

In three studies, aggressiveness of PCa was evaluated using the Gleason score via diagnostic biopsy. These three studies show strong association with prognosis. Risks associated with night shift work were higher among aggressive tumors as compared to less aggressive tumors [12]. Men who has never worked at nights and in night shifts do not have a lower risk of PCa in comparison with night-shift workers [12]. It is possible to suggest that poor prognosis PCa patients have lower melatonin level.

Modern treatment options available in the oncologist’s arsenal do not always make it possible to successfully fight with a poor prognosis of PCa, especially in the presence of a metastatic lesion or a low-grade tumor. It is in this category of patients that we observe the implementation of a multi-faceted mechanism of antitumor and immunomodulatory activity of melatonin. Numerous disturbances of homeostasis, angiogenesis, cell-to-cell communication and apoptosis observed in patients with poor prognosis are targets for melatonin.

The results of our study are in agreement with the data on favorable antitumor effect melatonin treatment of patients with advanced cancer of various localization, mainly in the breast, uterus, colon, etc., [1, 19–22]. Taking into consideration the optimistic conclusions obtained in our work, based on large clinical material, it is advisable to conduct prospective randomized studies with an assessment of survival rates in patients with PCa of various prognosis groups.

## MATERIALS AND METHODS

The retrospective study included 955 patients with prostate cancer who received combined hormone and radiation treatment at the Granov Russian Research Center for Radiology and Surgical Technologies from 2000 to 2019. The patients were included in the study based on the following criteria: there was a complete set of data on the outpatient examination, treatment and its results; and the patients were under dynamic observation until the date of death/cut-off date (01.06.2020), whichever came first.

All patients were diagnosed via transrectal prostate biopsy, followed by morphological verification and assessment of the Gleason scale scores. Patient staging was performed in accordance with the TNM classification recommended by the AJCC in 1997.

All patients were divided into two groups depending on their melatonin intake: research group (n-396, took melatonin per os, according to the scheme, for a long time); and control group (n-559, did not take melatonin). Melatonin was prescribed at the end of the course of remote radiotherapy or immediately after the diagnosis in the case of hormone therapy or chemotherapy. Melatonin was prescribed in tablet form, in a dosage of 3 mg, to be taken 30 minutes before sleep daily. Patients did not receive melatonin during the winter season (December-February). It is well known that in the winter season the melatonin level is at its maximum and in the spring–summer season the melatonin level is at its minimum [13].

In order to determine the predictive effect of melatonin intake on the survival rates of patients with prostate cancer, all patients were divided into prognostic groups. We used the D’Amico classification as a basis, which allowed us to form three prognostic groups: a favorable forecast (T1-T2a and GS ≤ 6 and PSA ≤ 10); an intermediate forecast (T2b and/or GS = 7 and/or PSA > 10–20); and poor forecast (≥ T2c or PSA > 20 or GS 8–10). Patients with radiologically confirmed metastatic PCa were included in the group of poor prognoses.

All patients received combined hormone radiation treatment. The treatment took into account the prevalence of the tumor process, and was in accord with the treatment protocols adopted for the period of antitumor therapy. External beam radiotherapy (EBRT) was performed on the linear accelerators of electrons with limit energy 6 to 18 MeV. Patients with favorable and intermediate prognosis underwent local radiation therapy on the prostate and seminal vesicles of a single local dose of 3 Gy, with the total local dose of 54–57 Gy (equivalent dose - 66–72 Gy). Poor prognosis PCa patients underwent EBRT by steps with the daily radiation dose. Patients with clinical positive lymph nodes at the first step of treatment received regional beam therapy with single 2 Gy doses up to total 40–44 Gy dose. The next step was based on local radiation. Patients with metastatic pelvic bones involvement were treated with beam therapy starting with segmental radiation step, with single 2 Gy doses up to 20 Gy total, afterwards regional and local radiation was performed according to the regimens mentioned above. Patients with generalized metastatic skeletal involvement accompanied with pain syndrome received systemic radiation therapy with 89Sr-chloride. Androgen deprivation therapy (ADT) was given to patients with various gonadotropin releasing hormone analogues and antiandrogen medications. Some patients underwent bilateral orchiectomy as hormone therapy method. Additional information on the treatment received by each prognosis group is contained in Tables 1–3.

The treated patients were monitored dynamically at three-month intervals during the first year, then every six months. We analyzed the overall survival rate (OS), which was calculated from the time of diagnosis to the date of the last observation or death of the patient from any cause.

For statistical analysis, the software package MedCalc 14.12.0 (MedCalc Software, Belgium) was used. To characterize interval variables which have a normal distribution, the mean (M) and standard deviation (s) were used. To characterize ordinal and interval variables whose distribution was not normal, the median (Me) and interquartile ranges (IQR) were estimated.

The T-criteria were used to evaluate differences in groups with normal distribution of the trait. To evaluate differences between two groups in the absence of an approximate normal distribution of the trait, the Mann–Whitney *U* test was used. To compare more than two independent samples, the Kruskal–Wallis variance analysis was applied. To assess the relationship of qualitative features, we used the cross-tabulation method (Pearson's Chi-square criterion, risk and chance assessment). In order to analyze the survival rate of patients with PCa, we performed the Kaplan-Meyer method of multiplying estimates, and the log – rank test was used for its comparison. When assessing the impact of a set of factors on survival, a regression model of proportional Cox risks was used. The significance level *p* < 0.05 was considered to be the criterion of statistical reliability of the obtained conclusions.
