# Addendum: Melatonin increases overall survival of prostate cancer patients with poor prognosis after combined hormone radiation treatment

**DOI:** 10.18632/oncotarget.28363

**Published:** 2023-02-25

**Authors:** Gennady M. Zharinov, Oleg A. Bogomolov, Irina V. Chepurnaya, Natalia Yu. Neklasova, Vladimir N. Anisimov

**Affiliations:** ^1^A.M. Granov Russian Research Center for Radiology and Surgical Technologies of the Ministry of Health of the Russian Federation, Pesochny, St. Petersburg 197758, Russia; ^2^N.N. Petrov National Medical Research Center of Oncology, Pesochny, St. Petersburg 197758, Russia

**This article has an addendum:** Patient consent was required and obtained. This study was approved by the Russian Research Centre of Radiology and Surgical Technologies Ethics Committee (Approval #1041Y).

Original article: Oncotarget. 2020; 11:3723–3729. 3723-3729. https://doi.org/10.18632/oncotarget.27757

